# Supplementary material for: Learning via mechanosensitivity and activity in cytoskeletal networks
Source: ArXiv. 2025 Apr 21:arXiv:2504.15107v1. Preprint. [Version 1] (PMC12045384)
Supplement: Supplement 1 [file NIHPP2504.15107v1-supplement-1.pdf]

Appendix A: Model Details

To understand how physical learning in a cytoskeletal network may occur, we consider the cytoskeletal network as a disordered network of nodes connected by edges. To create the initial disordered networks, we use an energy-minimized and force-balanced polydisperse particle mixture (with small and big particles in the $1:1$ ratio) with harmonic interaction. The networks are obtained by creating a Voronoi tessellation of the particle positions. The initial network is assumed to be tension-free, i.e., the length of each edge (L) is the same as their equilibrium length (L^0). We describe the dynamics of the network by the dynamics of the positions of the nodes $\{\boldsymbol{r}\}$ which governs the length of the edge L and the density of the bound motor m and the bound mechanosensitive protein n at each edge. We define the strain at the edge as $\epsilon = (L - L^0)$. We shall elaborate on the linearization used here by considering the dynamics of one edge in the network.

We consider a force dependent dynamics for the number density of bound mechanosensitive proteins given by

$$\dot{n} = k_{bn} - k^0_{un} e^{-\beta\dot{\epsilon}} n \,, \tag{A1}$$

where k_{bn} and k^0_{un} are the bare binding and unbinding rate and β is the coefficient for strain-rate dependent unbinding. Mechanosensitive protein dynamics can be very fast with a timescale of a few seconds. Here we consider the strain rate change to be a much slower process than the protein dynamics (i.e., consider constant strain rate when solving for n). Now we can linearize the dynamics around a steady state value n^0 and zero strain $\epsilon^0 = 0$ and solve for the variation δn as

$$\begin{aligned}\delta\dot{n} &= k_{bn} - k^0_{un}(1 - \beta\delta\dot{\epsilon})(n^0 + \delta n) \\ &= \beta n^0 k^0_{un}\delta\dot{\epsilon} - k^0_{un}\delta n \\ \delta n &= n^0\beta\delta\dot{\epsilon} + C_0 e^{-k^0_{un}t}\end{aligned} \tag{A2}$$

where $n^0 = k_{bn}/k^0_{un}$ is the unstrained steady state mechanosensitive protein density and C_0 is the constant of integration.

The molecular motor binding-unbinding kinetics is known to be mechanosensitive. Here, we consider a mechanosensitive protein-dependent bound motor dynamics given as

$$\dot{m} = k^0_b + k^1_b n - k_u m \tag{A3}$$

where k^0_b, k^1_b and k_u are the bare binding and unbinding rates. Now rewriting this above equation in terms of the motor density variation around the steady state value $m^0 = (k^0_b + k^1_b n_0)/k_u$ as $m = m^0 + \delta m$ and linearizing the terms we get

$$\begin{aligned}\delta\dot{m} &= k^0_b + k^1_b(n_0 + \delta n) - k_u(m_0 + \delta m) \\ &= k^1_b\delta n - k_u\delta m \\ &\simeq k^1_b\beta n_0\delta\dot{\epsilon} - k_u\delta m \,,\end{aligned} \tag{A4}$$

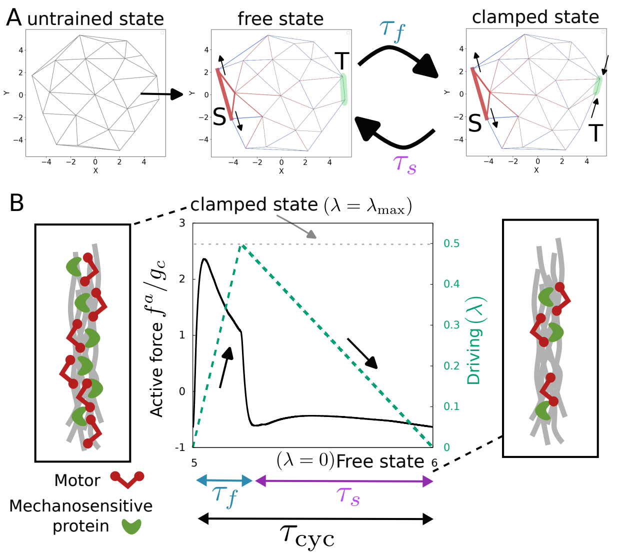

FIG. S1. Contrastive learning via driving. (A) The network in free and clamped states. The thickness indicates strain at each edge and the color indicates contraction (blue) or extension(red). (B) Representative active force dynamics (due to change in motor density) in one edge as the target edge is driven from the free to the clamped state over a fast timescale τ_f and brought back to the free state over a slow timescale τ_s. The motor and mechanosensitive protein density increases when the edge is extended in the clamped state. The figure shows unitless quantities and the rescaled parameter values used in panel B are the same as Fig. 2.

here we use the steady-state protein variation (as the protein dynamics is fast) to arrive at the above equation.

The dynamics of the learning degree of freedom (i.e., the rest length in this case) of an edge depends on the active force (f^a) on that edge which is a function of the motor density variation in the edge and it is given by

$$\dot{L}^0_i = \alpha g(f^a) \,, \tag{A5}$$

where α is the learning rate and $g(x) = x$ for all $|x| \geq g_c$ and 0 otherwise. The learning in this model is continuous and driven by forcing the system from the free state to the clamped state fast and bringing back slowly over two timescales τ_f and τ_s respectively.

Now, the dynamics of the jth node and the adjacent edge between nodes j and k can be described in terms of node position and the variation of motor density as:

$$\begin{aligned}\gamma\dot{\boldsymbol{r}}_j &= \sum_k^{nn} -k_{jk}(|\boldsymbol{r}_j - \boldsymbol{r}_k| - L^0_{jk})\hat{\boldsymbol{r}}_{jk} + f^a_{jk}\hat{\boldsymbol{r}}_{jk} \\ \delta\dot{m}_{jk} &= k^1_b\beta n_0\delta\dot{\epsilon}_{jk} - k_u\delta m_{jk} \\ \dot{L}^0_{jk} &= \alpha g(f^a_{jk})\end{aligned} \tag{A6}$$

where L^0_{jk} and k_{jk} are the instantaneous rest length and the stiffness of the edge. The active force in the bond

is given by $f^a_{jk} = \xi \delta m_{jk}$. We shall use the disordered network with the above-described dynamics to study physical learning in cytoskeletal networks.

Rescaled dynamical equations

To derive a set of rescaled dynamical equations, we consider the average distance between the nodes in a network as a length scale l and the duration of a driving cycle $\tau_{\rm cyc} = \tau_f + \tau_a$ as a time scale. We can rewrite the rescaled dynamical equations in terms of unitless parameters as

$$\begin{aligned}\dot{\tilde{\boldsymbol{r}}}_j &= \sum_k^{nn} -\tilde{k}_{jk}(|\tilde{\boldsymbol{r}}_j - \tilde{\boldsymbol{r}}_k| - \tilde{L}^0_{jk})\hat{\boldsymbol{r}}_{jk} + \tilde{f}^a_{jk}\hat{\boldsymbol{r}}_{jk} \\ \delta\dot{\tilde{m}}_{jk} &= \tilde{\beta}\delta\dot{\tilde{\epsilon}}_{jk} - \tilde{k}_u\delta\tilde{m}_{jk} \\ \dot{\tilde{L}}^0_{jk} &= \tilde{\alpha}g(\tilde{f}^a_{jk}) \end{aligned} \qquad \text{(A7)}$$

where $\tilde{\boldsymbol{r}} = \boldsymbol{r}/l$, $\delta\tilde{m}_{jk} = \delta m_{jk}/m_0$, $\tilde{L}^0_{jk} = L^0_{jk}/l$ and $\tilde{f}^a_{jk} = \tilde{\xi}\tilde{m}_{jk}$. The unitless parameters are defined as $\tilde{k}_{jk} = \frac{k\tau_{\rm cyc}}{\gamma}$, $\tilde{\xi} = \frac{\xi m_0 \tau_{\rm cyc}}{\gamma l}$, $\tilde{\beta} = \frac{\beta k^1_b n_0 l}{m_0}$, $\tilde{k}_u = k_u\tau_{\rm cyc}$ and $\tilde{\alpha} = \frac{\alpha\tau^2_{\rm cyc}\xi}{\gamma l^2}$. We shall drop the ˜ sign in the discussions to keep notations simple. Here the motor density is rescaled by an equilibrium motor density m_0. The average distance in the networks we used is $l \simeq 2.5\,\mu m$. Here, the kernel timescale is determined by the motor turnover timescale $\tau_k = k_u^{-1}$ (see Appendix B) and molecular motors like the myosin motor have turnover timescale of a few seconds. For learning via driving at the target, the timescale of driving should be much larger than the timescale of the memory kernel ($\tau_k < \tau_f < \tau_s$) [58]. Hence, we consider a cycle duration much larger than motor turnover timescale $\tau_{\rm cyc} = 100\,\text{sec}$ with $\tau_f/\tau_s = 1/4$ unless otherwise specified. In the case of classification of environmental signals and self-organized learning we have used $\tau_{\rm cyc} = 10\,sec$ and $\tau_{\rm cyc} = 50\,sec$ correspondingly. We consider an equilibrium motor number density $m_0 = 10\,\mu m^{-1}$ and the factor $k^1_b n_0 = 1\,\mu m^{-1}\text{sec}^{-1}$. We shall use these described values to derive a set of unitless parameters for our analysis. The threshold value g_c in the activation function $g(x)$ is a hyper-parameter of learning and set at an optimal value in the range of $10^{-6} - 10^{-5}$.

Supervised learning

Here we consider a learning scheme based on the above-described supervised temporal contrastive learning method where the supervisor controls the driving mechanism in which the system is taken from the free state to the clamped state and back according to the desired behaviour (strain at the target edge in this case). This driving force at the target nodes can be described as

$$\boldsymbol{f} = \lambda(t)\boldsymbol{\nabla}(\frac{\lambda}{2}|\epsilon_T - \epsilon^*_T|^2) \qquad \text{(A8)}$$

where ϵ_T and ϵ^*_T are instantaneous and desired strain at the target edge. The function $\lambda(t)$ is a sawtooth function devised according to previously used driving in Falk et al [58]. It controls the timescale of driving by incorporating a fast transition (over a time duration τ_f) from free to clamped state and a slow relaxation (over a time duration τ_s) back to the free state.

Appendix B: Memory kernel

The motor dynamics coupled with the mechanosensitive proteins, possesses the memory of local strain. We can write the motor density variation in the integral form and use the integration by parts to show

$$\begin{aligned}\delta m &= \beta_1 \int_{-\infty}^{t} e^{-\frac{(t-t')}{\tau_k}}\delta\dot{\epsilon}(t')dt' \\ &= \beta_1\delta\epsilon - \frac{\beta_1}{\tau_k}\int_{-\infty}^{t} e^{-\frac{(t-t')}{\tau_k}}\delta\epsilon(t')dt' \\ &= \int_{-\infty}^{t}\mathcal{K}(t-t')\delta\epsilon(t')dt' \end{aligned} \qquad \text{(B1)}$$

where the memory kernel is given by

$$\mathcal{K}(t-t') = \beta_1\left(\delta(t-t') - \frac{1}{\tau_k}e^{-\frac{(t-t')}{\tau_k}}\right). \qquad \text{(B2)}$$

The consistency between the integral form and the motor dynamics described in Eq. A6 can be understood if we take a derivative of the above integral form (Eq. B1) and use Leibniz's integral rule

$$\begin{aligned}\delta\dot{m} &= \beta_1\frac{d}{dt}\left[\int_{-\infty}^{t} e^{-\frac{(t-t')}{\tau_k}}\delta\dot{\epsilon}(t')dt'\right] \\ &= \beta_1\frac{d}{dt}\left[\int_{-\infty}^{t} f(t,t')dt'\right] \\ &= \beta_1 f(t,t')\Big|_t \frac{d}{dt}(t) - \beta_1 f(t,t')\Big|_{-\infty}\frac{d}{dt}(-\infty) \\ &\quad +\beta_1\int_{-\infty}^{t}\frac{\partial}{\partial t}\left(f(t,t')\right) \\ &= \beta_1\delta\dot{\epsilon}(t) - \frac{\beta_1}{\tau_k}\int_{-\infty}^{t} e^{-\frac{(t-t')}{\tau_k}}\delta\dot{\epsilon}(t')dt' \\ &= \beta_1\delta\dot{\epsilon}(t) - \frac{1}{\tau_k}\delta m \end{aligned} \qquad \text{(B3)}$$

which we can now compare with the motor dynamics (Eq. A6) to identify the parameters $\tau_k = k_u^{-1}$ and $\beta_1 = k^1_b\beta n_0$. In the rescaled parameters, the memory kernel parameters will be slightly different given by $\tilde{\beta}_1 = \tilde{\beta}$ and $\tilde{\tau}_k = \frac{1}{k_u\tau_{\rm cyc}}$.

Appendix C: Learning in nonlinear regime

We have taken a linear approximation in the mechanosensitive protein to derive a motor-dependent

implicit memory of local strain. We find that this linear approximation is not a necessary condition for learning. Here we consider higher-order terms in the protein dynamics given by Eq. 3 in the maintext to arrive at

$$\begin{aligned}\delta\dot{n} &\simeq k_{bn} - k^0_{un}\left(1 - \beta\delta\dot{\epsilon} + \frac{\beta^2}{2}\delta\dot{\epsilon}^2 - \frac{\beta^3}{6}\delta\dot{\epsilon}^3\right)(n_0 + \delta n) \\ &= \beta n_0 k^0_{un}\phi(\delta\dot{\epsilon}) - k^0_{un}\delta n \end{aligned} \tag{C1}$$

where $\phi(\delta\dot{\epsilon}) = \delta\dot{\epsilon} - \frac{\beta}{2}\delta\dot{\epsilon}^2 + \frac{\beta^2}{6}\delta\dot{\epsilon}^3$. We have ignored the contributions of mixed terms (e.g. $\delta n\delta\dot{\epsilon}$) for analytical tractability. Similar to Appendix. A, we can obtain the solution for protein dynamics given by

$$\delta n = n_0\beta\phi(\delta\dot{\epsilon}) + C'_0 e^{-k^0_{un}t}\,, \tag{C2}$$

where C'_0 is a constant of the integration.

Now, using the steady state solution of protein density variation dynamics given above and the Eq. A4, we arrive at motor density variation dynamics given by

$$\begin{aligned}\delta\dot{m} &= k^1_b\delta n - k_u\delta m \\ &\simeq k^1_b\beta n_0\phi(\delta\dot{\epsilon}) - k_u\delta m \\ &= \beta_1\delta\dot{\epsilon} - \beta_2\delta\dot{\epsilon}^2 + \beta_3\delta\dot{\epsilon}^3 - k_u\delta m\,, \end{aligned} \tag{C3}$$

where $\beta_1 = k^1_b n_0\beta$, $\beta_2 = k^1_b n_0\beta^2$ and $\beta_3 = k^1_b n_0\beta^3$.

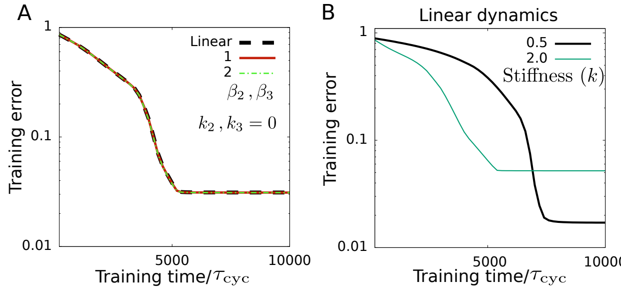

FIG. S2. Learning with non-linear mechanosensitive protein dynamics. (A) The temporal evolution of training error does not significantly change in presence of moderate non-linearity. (B) Training error time evolution indicates better learning when the network edges are softer. The parameter values used here are the same as Fig. 2 except $\lambda_{max} = 0.2$ and $\xi = 0.2$.

To understand the implicit memory in the motor dynamics we consider the integral form given by

$$\begin{aligned}\delta m &= \beta_1\int_{-\infty}^{t} e^{-\frac{(t-t')}{\tau_k}}\left(\delta\dot{\epsilon} - \frac{\beta}{2}\delta\dot{\epsilon}^2 + \frac{\beta^2}{6}\delta\dot{\epsilon}^3\right)dt' \\ &= I_1 + I_2 + I_3 \end{aligned} \tag{C4}$$

where the integral I_1 is same as described in Eq. B1 and integrals I_2 and I_3 can be written as

$$I_2 = -\frac{\beta}{2}\int_{-\infty}^{t}\mathcal{K}(t-t')h(t')dt' \tag{C5}$$

and

$$I_3 = \frac{\beta^2}{6}\int_{-\infty}^{t}\mathcal{K}(t-t')p(t')dt' \tag{C6}$$

where $h(t) = \int_{-\infty}^{t}\delta\dot{\epsilon}^2 dt$ and $p(t) = \int_{-\infty}^{t}\delta\dot{\epsilon}^3 dt$. We approximate these integrals as

$$\begin{aligned}h(t) &= \int_{-\infty}^{t}\delta\dot{\epsilon}^2 dt \\ &\simeq \int_0^{\epsilon}\delta\dot{\epsilon}d\epsilon \\ &\simeq \int_0^{\epsilon}\frac{\epsilon}{\tau}d\epsilon \\ &= \frac{\epsilon^2}{2\tau} \end{aligned} \tag{C7}$$

where we approximate the strain rate $\sim \frac{\epsilon}{\tau}$ using a characteristic timescale τ. We can rewrite the integral form of motor density variation as

$$\delta m = \int_{-\infty}^{t}\mathcal{K}(t-t')\left(\epsilon(t') - \frac{\beta}{4\tau}\epsilon^2(t') + \frac{\beta^2}{18\tau^2}\epsilon^3(t')\right)dt' \tag{C8}$$

which shows motor density has an implicit memory of a nonlinear function of local strain rather than simply strain. The learning mechanism still works with the same learning rule even though the motor dynamics does not accurately estimate the difference in strain between the free and the clamped state with the above-described form. The training error indicates no significant change in the non-linear regime compared to the linear approximation (Fig. S2A).

Cytoskeletal networks are known to have nonlinear elasitcity. We consider an elastic energy with higher order terms given by

$$E_{el} = \frac{1}{2}k\epsilon^2 + \frac{1}{3}k_2\epsilon^3 + \frac{1}{4}k_3\epsilon^4 \tag{C9}$$

where k_2 and k_3 are elastic constants corresponding to the higher order terms. The cubic term in the elastic energy originates from activity in the cytoskeletal networks and can take both signs and the quartic term indicates effects like strain-stiffening. Considering nonlinear elasticity, make the contrastive update [57, 58] depend on a nonlinear function of strain

$$\begin{aligned}\dot{L}_0 &= \left(\frac{\partial E_{el}}{\partial L_0}\right|_{\rm free} - \left(\frac{\partial E_{el}}{\partial L_0}\right|_{\rm clamped} \\ &= \left(k\epsilon + k_2\epsilon^2 + k_3\epsilon^3\right)|_{\rm clamped} \\ &\quad - \left(k\epsilon + k_2\epsilon^2 + k_3\epsilon^3\right)|_{\rm free}\,. \end{aligned} \tag{C10}$$

Hence, the implicit memory of a nonlinear function of strain in motor dynamics can now enable contrastive

learning for nonlinear elastic interactions. Together with the non-linearities in mechanosensitive protein dynamics, we find the strength of nonlinearity to affect learning and with increasing strength of non-linearity the training error becomes smaller (Fig. 3D). The network softening due to the cubic nonlinearity may play a role in affecting learning as a softer network learns better and reaches lower training error (Fig. S2B).

SUPPLEMENTARY FIGURES

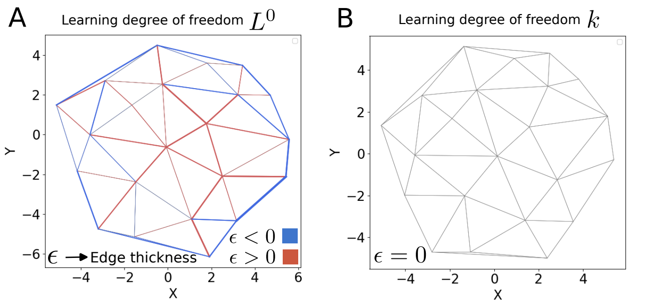

FIG. S3. Strain in the trained network. (A) Strain in a trained network with the rest length (L^0) as the learning degree of freedom. The edge thickness and colour indicate local strain magnitude and sign. (B) A trained network with the edge stiffness (k) as the learning degree of freedom has no strain in the equilibrium state (i.e., without any source strain). The parameters are same described in Fig. 2 and Fig. 3.

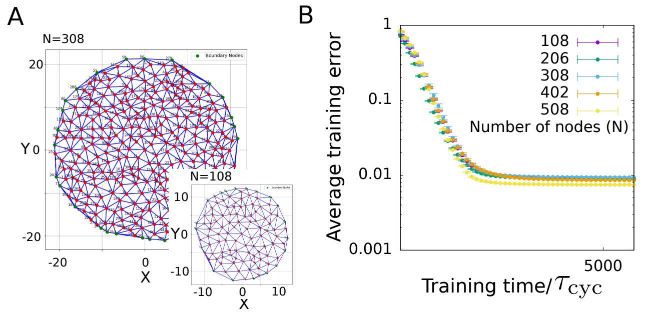

FIG. S4. Effect of network size on learning. (A) Networks of different sizes are used for the learning task. One source and one target edge were randomly chosen from the edges on the network periphery. (B) Training error with training time for different sizes shows no significant effect of network size on learning. The parameters used here are the same as Fig. 2 except $\tau_{\rm cyc} = 800\,sec$.

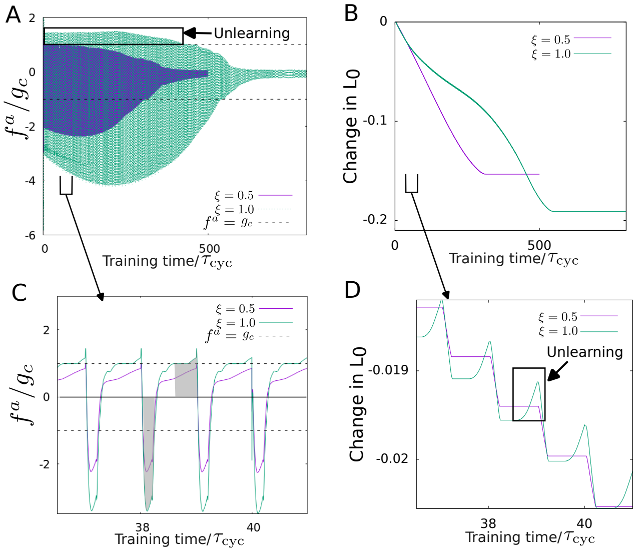

FIG. S5. Effect of activity on learning at a single edge. (A) Rescaled active force with time during training in one specific edge of the network. Higher activity shows longer and more learning but also has a significant period of unlearning (highlighted). (B) Higher activity leads to an increased change in rest length (L^0) of the edge during training. (C) A zoomed-in version of rescaled active stress vs training time shows the difference in active force at different activity values. The shaded region shows learning (when $f^a < 0$) and unlearning (when $f^a > 0$). (D) A zoomed-in version of change in rest length during training shows a larger amount of learning in each cycle and a large amount of unlearning at a higher activity value. The parameters used here are the same as Fig. 2.

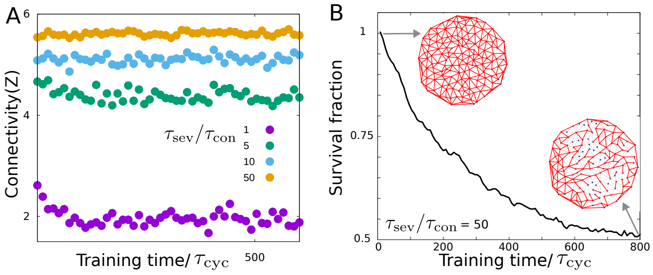

FIG. S6. Learning with turnover of network edges. (A) Average network connectivity at different $\tau_{\rm sev}/\tau_{\rm con}$ ratio during training. (B) Fraction of all edges that have not been severed at any time during the training (survival fraction). Insets show the network at the beginning and ending of training. The edges that have never been severed are indicated in red. Parameter values used here are the same as Fig. 5.
